# Genomic characterization of parvovirus and beak and feather disease virus in cockatiel (*Nymphicus hollandicus*)

**DOI:** 10.1128/mra.00820-24

**Published:** 2024-10-29

**Authors:** Subir Sarker, Saranika Talukder, Md. Safiul Alam Bhuiyan, Md. Hakimul Haque

**Affiliations:** 1 Biomedical Sciences & Molecular Biology, College of Public Health, Medical and Veterinary Sciences, James Cook University, Townsville, Queensland, Australia; 2 Australian Institute of Tropical Health and Medicine, James Cook University, Townsville, Queensland, Australia; 3 Department of Microbiology, Anatomy, Physiology, and Pharmacology, School of Agriculture, Biomedicine and Environment, La Trobe University, Melbourne, Victoria, Australia; 4 College of Public Health, Medical and Veterinary Sciences, James Cook University, Townsville, Queensland, Australia; 5 Faculty of Sustainable Agriculture, Livestock Production, University Malaysia Sabah, Sandakan, Sabah, Malaysia; 6 Department of Veterinary and Animal Sciences, University of Rajshahi, Rajshahi, Bangladesh; Katholieke Universiteit Leuven, Leuven, Belgium

**Keywords:** parvovirus, beak and feather disease virus, avian

## Abstract

This study reveals the genomes of psittaciform chaphamaparvovirus 5 (PsChPV-5) and a beak and feather disease virus (BFDV), discovered in the fecal samples of cockatiels. The genomes of PsChPV-5 and BFDV are 4,366 and 2,009 base pairs long, respectively, each exhibiting the characteristic genomic structures of their respective genera.

## ANNOUNCEMENT

Chaphamaparvoviruses (ChPVs), part of the *Parvoviridae* family, are nonenveloped, icosahedral viruses with a 4.0 to 4.5 kb linear single-stranded DNA genome (1). They have two major genes: a nonstructural (NS1) replicase and a capsid (VP) gene (2, 3). ChPVs, widespread in nature, have been found in the feces of birds (4–8) and mammals (9) and linked to renal disease in lab mice (10). Recently, ChPVs were detected in the liver of rainbow lorikeets (11) and chickens (12, 13) in Australia. Beak and feather disease virus (BFDV), from the *Circoviridae* family, has a 2.0 kb circular single-stranded DNA genome (14) and infects various Australian psittacine (15, 16) and non-psittacine birds (17–20). This study reports a PsChPV-5 and a BFDV genomes in cockatiels (*Nymphicus hollandicus*).

Fecal samples were collected from a group of healthy captive cockatiels (*n* = 4) housed in a cage at a pet shop in Victoria, Australia (37°1′12.36″S, 144°57′52.56″E) during routine care without handling the birds. The Animal Ethics Committee at La Trobe University was informed that findings from the material (with no bird touching) were to be used in a publication, and a formal waiver of ethics approval was granted. The samples were enriched for viral particles as described before (21), followed by viral nucleic acids extraction using the QIAamp viral RNA minikit (Qiagen, USA) without carrier RNA, allowing for simultaneous DNA and RNA extraction. Prior to library construction, the extracted nucleic acids was subjected to cDNA synthesis, and amplification was conducted using the Whole Transcriptome Amplification Kit (WTA2, Sigma-Aldrich, Darmstadt, Germany) following the manufacturer’s instructions. Library was prepared using the Illumina DNA Prep (Illumina, San Diego, USA) as per kit instructions, starting with 250 ng of purified DNA (6). The Australian Genome Research Facility (AGRF) in Melbourne evaluated the library quality and sequenced it on the Illumina NovaSeq platform, producing 150 bp paired-end reads.

Sequencing data were processed as per established pipeline (22–25) using Geneious Prime (version 2023.1.1, Biomatters, New Zealand). Initially, 31.99 million raw reads were pre-processed to remove the Illumina adapter, ambiguous base calls, and poor-quality reads (trim using quality score, limit 0.05; trim ambiguous nucleotide up to 15), followed by mapping against the chicken genome (*Gallus gallus*, GenBank accession no. NC_006088) to exclude host DNA. Subsequently, 31.86 million trimmed, unmapped reads were assembled *de novo* using SPAdes assembler (version 3.15.5) in Geneious Prime, generating a 4,366 bp PsChPV-5 genome (average coverage 165.74×) and a 2,009 bp BFDV genome (average coverage 23.18×). Genome annotation was performed using default parameters under the standard genetic code (transl_table 1) in Geneious Prime. All software was used with default parameters except where stated.

**Fig 1:**
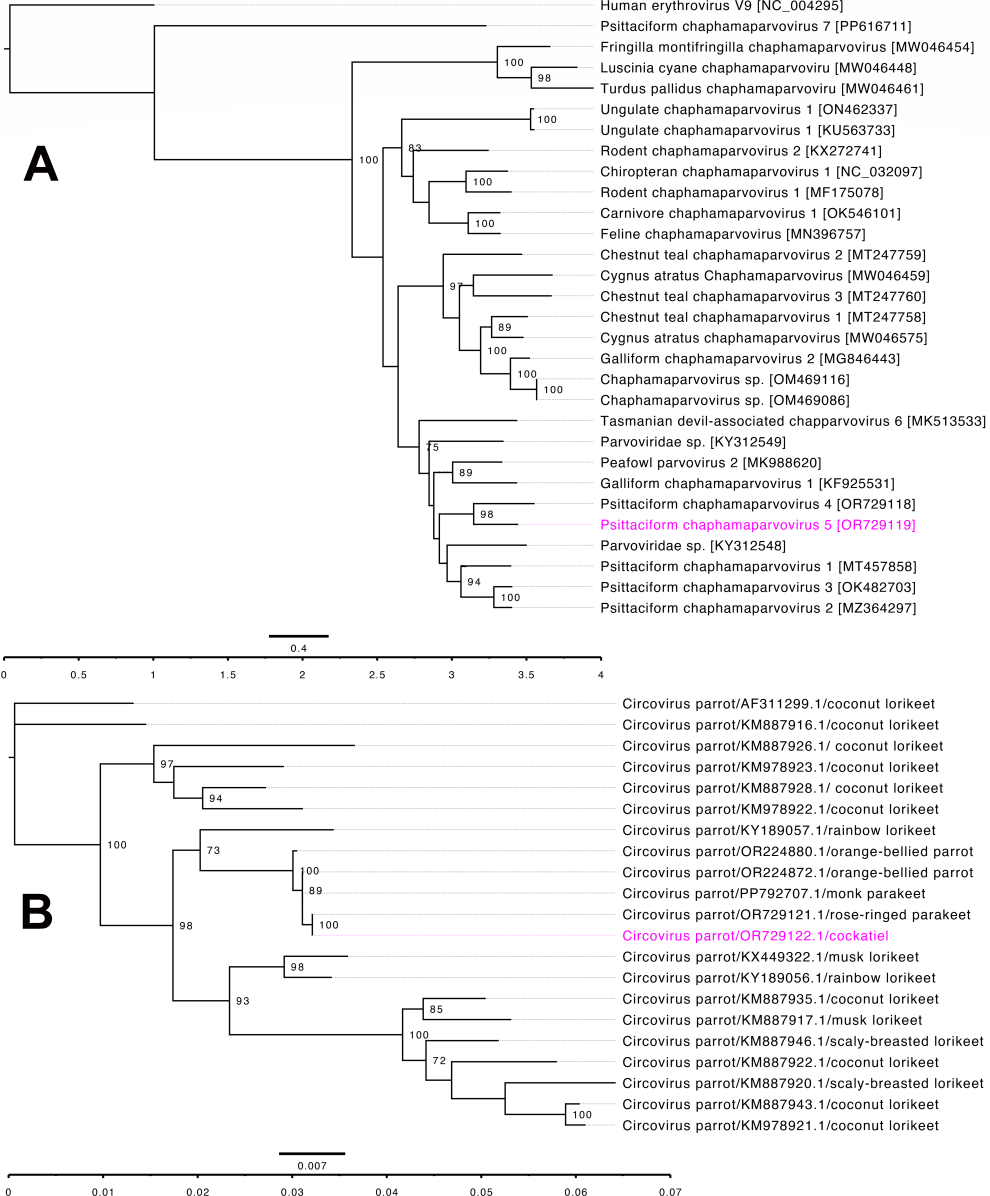
The phylogenetic tree illustrates the potential evolutionary relationships of selected parvoviruses (**A**) and BFDV (**B**). (**A**) Amino acid sequences of complete NS1 gene were extracted individually from the PsChPV-5 and various selected parvovirus genomes and then aligned with MAFTT (version 7.450), using G–INS–I (scoring matrix BLOSUM62; gap open penalty 1.53; offset value 0.123) in Geneious Prime (version 23.1.1, Biomatters, Ltd., Auckland, New Zealand). Maximum likelihood (ML) tree was generated in Geneious Prime with 500 replicates using human erythrovirus V9 as the outgroup. Sequence diversities are indicated as substitutions per site to the branches, and the labels at the branch tips represent the original parvovirus species names along with their GenBank accession numbers in parentheses. The PsChPV-5 sequence analyzed in this study is highlighted in pink. (**B**) Selected complete genome sequences of BFDV were aligned with MAFTT (version 7.450), using G–INS–I in Geneious Prime (version 23.1.1). Maximum likelihood (ML) tree was generated in Geneious Prime with 500 replicates. Trees were visualized suing FigTree v1.4.4 and tips labels were aligned. Sequence genetic distance are indicated as substitutions per site to the branches in branch labels, and the labels at the branch tips represent the representative virus species names along followed by their GenBank accession and host. The BFDV sequence analyzed in this study is highlighted in pink. Automatic scale bar and scale axis were added. Bootstrap values at the nodes are indicated as percentages (bootstrap value lower than 70% was removed from the trees).

The PsChPV-5 genome contained four open reading frames (ORFs), whereas the BFDV genome contained two, as expected according to their viral genera. Comparative analysis of the predicted ORFs were conducted by using BLASTX and BLASTP (26) (Table 1). The genomes of PsChPV-5 and BFDV showed the highest nucleotide identity (using BLASTn) with a parvovirus sequenced from a Nanday parakeet (73.77% identity, 39% query coverage; GenBank accession number MW046381.1) and BFDV sequenced from an orange-bellied parrot (99.55% identity, 100% query coverage; GenBank accession number OR224872.1), respectively. Phylogentically, PsChPV-5 shows the strongest relationship with parvoviruses from parrots (Fig. 1A). Similarly, BFDV sequenced in this stdudy clustered with BFDV sequenced from various host species including the orange-bellied parrot in Australia (Fig. 1B).

**TABLE 1:** Summary of the detected viruses^*a*^

Virus name	GenBank accession	Genome length/completeness	G + C content (%)	% of Top BLAST hit (GenBank accession/virus name/infected host)				% of Top BLAST hit (GenBank accession/virus name/infected host)	
				NS1	NS2	NS3	VP1	Replication-associated (Rep) gene	Capsid gene (Cap)
PsChPV-5	OR729119	4,366 nt, no (however, all the coding genes are complete)	42.5	54.09%, (WOX03037.1/*Psittaciform chaphamaparvovirus* 4/rose-ringed parakeet)	62.98% (WOX03039.1/*Psittaciform chaphamaparvovirus* 4/rose-ringed parakeet)	54.23% (WOX03049.1/*Psittaciform chaphamaparvovirus* 6/*Alexandrine parakeet*)	52.64%, (WOX03047.1/*Psittaciform chaphamaparvovirus* 6/*Alexandrine parakeet*)	NA	NA
BFDV	OR729122	2,009 nt, yes	54.1	NA	NA	NA	NA	100%, (WOX03051.1/BFDV/rose-ringed parakeet)	100%, (WOX03052.1/BFDV/rose-ringed parakeet)

^*a*^ NA = particular gene does not belong to the specific virus.

Like other parvoviruses, the complete NS1 gene of PsChPV-5 was 669 amino acids in length and encodes the helicase, including the conserved ATP- or GTP-binding Walker A loop (GPxNTGKT/S; _318_**GP**S**NTGKS**_325_), Walker B (xxxWEE; _357_IGV**WEE**_362_) Walker B’ (KQxxEGxxxxxPxK; _374_**KQ**VM**EG**MTTSI**P**V**K**_387_), and Walker C (PxxxTxN; _398_**P**IIV**T**T**N**_404_) aa motifs. In addition, the NS1 protein contains two conserved replication initiator (endonuclease) motifs, xxHuHxxxx (IF_108_**H**V**H**_110_VIYR) and YxxK (_166_**Y**LL**K**_169_) (conserved amino acids are indicated in bold letters, and “u” indicates a hydrophobic residue).

This study provides evidence of a parvovirus and a BFDV in healthy cockatiels, expanding the known host range of PsChPV and suggesting that some ChPVs may have a broader host spectrum.

## Data Availability

The complete viral genome sequences from this study have been deposited in DDBJ/ENA/GenBank under the accession numbers OR729119 (Link: https://www.ncbi.nlm.nih.gov/nuccore/OR729119) and OR729122 (Link: https://www.ncbi.nlm.nih.gov/nuccore/OR729122). The version described in this paper is the first version, OR729119.1 and OR729122.1. The raw sequencing data from this study have been deposited in the NCBI Sequence Read Achieve (SRA) under the accession number of SRR26413811 (Link: https://www.ncbi.nlm.nih.gov/sra/SRR26413811) and BioProject accession number: PRJNA1028305 (Link: https://www.ncbi.nlm.nih.gov/bioproject/PRJNA1028305).
